# Monitoring the Mind: The Neurocognitive Correlates of Metamemory

**DOI:** 10.1371/journal.pone.0030009

**Published:** 2012-01-05

**Authors:** Anne T. A. Do Lam, Nikolai Axmacher, Juergen Fell, Bernhard P. Staresina, Siegfried Gauggel, Tobias Wagner, Jan Olligs, Susanne Weis

**Affiliations:** 1 Department of Epileptology, University of Bonn, Bonn, Germany; 2 Department of Psychiatry and Psychotherapy, RWTH Aachen University, Aachen, Germany; 3 German Center of Neurodegenerative Diseases, Bonn, Germany; 4 MRC Cognition and Brain Sciences Unit, Cambridge, United Kingdom; 5 Department of Medical Psychology and Sociology, RWTH Aachen University, Aachen, Germany; 6 Helmholtz-Institute for Radiation and Nuclear Physics, University of Bonn, Bonn, Germany; 7 Department of Psychology, Durham University, Durham, United Kingdom; Katholieke Universiteit Leuven, Belgium

## Abstract

Memory performance in everyday life is often far from perfect and therefore needs to be monitored and controlled by metamemory evaluations, such as judgments of learning (JOLs). JOLs support monitoring for goal-directed modification of learning. Behavioral studies suggested retrieval processes as providing a basis for JOLs. Previous functional imaging research on JOLs found a dissociation between processes underlying memory prediction, located in the medial prefrontal cortex (mPFC), and actual encoding success, located in the medial temporal lobe. However, JOL-specific neural correlates could not be identified unequivocally, since JOLs were given simultaneously with encoding. Here, we aimed to identify the neurocognitive basis of JOLs, i.e., the cognitive processes and neural correlates of JOL, separate from initial encoding. Using functional magnetic resonance imaging (fMRI), we implemented a face-name paired associative design. In general, we found that actual memory success was associated with increased brain activation of the hippocampi bilaterally, whereas predicted memory success was accompanied by increased activation in mPFC, orbital frontal and anterior cingulate cortices. Masking brain activation during predicted memory success with activation during retrieval success revealed BOLD increases of the mPFC. Our findings indicate that JOLs actually incorporate retrieval processes.

## Introduction

Metamemory refers to the awareness and knowledge of our own memory. It includes the monitoring and control of memory processes [1], and is essential for their modification and optimization [2]. Even though the exact relationship between monitoring and control is still a matter of intense debate [3], [4], clearly both monitoring and control play an important role in a variety of everyday life situations. For instance, depending on monitoring results, a less effective learning strategy can be changed, the study of material which has not yet been mastered, can be repeated, or external cues can be employed to improve remembering [5], [6]. To ensure the efficacy of the metacognitive system, continuous feedback between monitoring and control mechanisms is required, which is provided by metacognitive judgments. Judgments of learning (JOLs) are one kind of such metacognitive judgments, which can be defined as prospective confidence judgments of encoding efficiency made after the acquisition of an item but prior to a recall test [7].

How JOLs are formed is still an open question. Behavioral studies hypothesized that JOLs are based, at least partially, on online-monitoring of the results of retrieval attempts of the target itself or target-related information [8]. On the other hand, a recent meta-analysis of the influence of JOLs demonstrated that metamemory predictions elicited only with the stimulus cue do not necessarily lead to improved performance in subsequent memory tests [9], as the monitoring retrieval hypothesis would predict. Thus, it might be questioned whether retrieval attempts actually constitute the basis for JOLs, or whether other factors, such as the imagery value of an item, might inform JOLs more reliably, as proposed in the cue-utilization approach of Koriat [10].

Neuroimaging research on the neural basis of JOLs was recently presented by Kao, Davis, and Gabrieli [11]. In this study, participants estimated during encoding whether they would later be able to recognize each presented item. Brain activation in the ventromedial prefrontal cortex increased with predicted retrieval success during encoding, whereas actual subsequent memory was associated with enhanced activity of the medial temporal lobes (MTL). This study provided a first approach to the investigation of the neural correlates of encoding-related monitoring processes. However, as JOLs were given simultaneously with stimulus encoding, the neural correlates of encoding and JOLs could not be disentangled unequivocally.

Furthermore, Kao and colleagues used an item recognition memory design. The criterion for JOLs in such a design is the distinctiveness of an item: if salient perceptual features are available, they increase the probability that the candidate item will be recognized as old [12].

In contrast, in associative memory designs, JOLs are based on the evaluation of memory representations in a more narrow sense. Such representations result from relational processes in which the critical stimuli and associated details are combined into a network of feature information [13].

Here, we were especially interested in associative memory, since in such designs, metamemory is based upon retrieval operations [8], [14], [15], for instance to get access to information associated to the critical item. In comparison to non-associative item recognition memory, associative memory has been shown to pertain to elevated activations in the hippocampus during memory formation [16]. Therefore, we implemented an associative learning design in which face-name pairs were presented for encoding. In order to clearly separate metamnemonic from encoding processes, JOLs were separated from encoding trials by a temporal delay.

We hypothesized that during JOLs, participants might run attempts to retrieve the target-related information or the target itself, and base their JOLs on the monitoring of the result of these attempts. On the basis of previous findings, we expect metamnemonic monitoring to be reflected by increased activation of medial prefrontal areas [11]. Moreover, if JOLs are actually based on the monitoring of retrieval processes, we reasoned that predicting successful memory performance and retrieval success itself should involve increased activation of at least partly overlapping brain networks.

## Methods

### Ethics Statement

This study was approved by the local ethical committee of the faculty of medicine of the RWTH University Hospital Aachen (“Ethik-Kommission an der Medizinischen Fakultät der Rheinisch-Westfälischen Technischen Hochschule Aachen (RWTH Aachen)”), according to the latest version of the Declaration of Helsinki, and all participants provided written informed consent.

### Participants

Seventeen native German speakers (eight female; mean age = 24.9 years), who were right-handed according to the Edinburgh inventory [17], participated in the study. All had normal or corrected vision, and reported no mental or neurologic disease. Data of eleven other participants had to be excluded from the final analysis due to floor and ceiling effects (5), technical problems (4), or excessive head motion during overt talk (2), respectively.

### Stimulus material

Stimulus material consisted of 130 colored full frontal photographs of faces provided by a face database designated especially for research purposes [18] (http://agingmind.cns.uiuc.edu/facedb/). The pictures displayed faces of male and female adults, exhibiting a neutral facial expression. Additionally, we used 130 German first names provided by a webpage on which the most popular first names per decade and per gender are registered (http://www.beliebte-vornamen.de). Both facial stimuli and first names were evaluated by 38 undergraduate students. The facial stimuli were rated with respect to age, emotional expression, and distinctiveness. The first name stimuli were rated with respect to familiarity, frequency, and unequivocality of gender. Finally, to match the age of the stimuli faces to the participantś age, a set of 30 male and 30 female faces which were rated as aged 18–29, as exhibiting a neutral facial expression and as lacking any distinct facial features were randomly assigned to first names scoring highly in the above mentioned evaluation criteria.

### Task procedure

**Figure 1:**
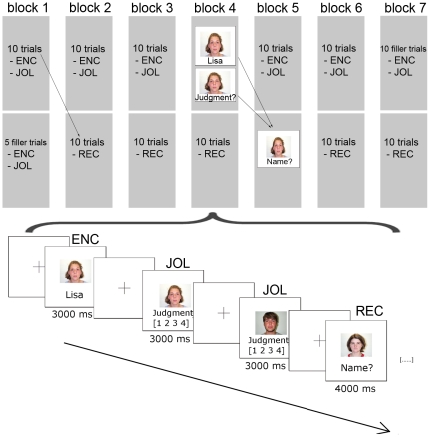
Experimental design. Overview of the experimental design which consisted of seven blocks. Encoding (ENC) and JOL trials in the first phase of each block were followed by a phase with recall trials. Sample encoding trials, JOL trials, and recall (REC) trials. Encoding and JOL trials were presented for 3000 ms, recall trials for 4000 ms, ISI varied from 2400 ms to 4200 ms. For each face with the caption “Judgment?”, participants provided a judgment of learning. For each face with the caption “Name?”, the previously presented name had to be recalled.

Participants were scanned while they were presented with a series of faces, which were paired with fictional first names (encoding trials). The task was to study the faces together with the corresponding name; no response was required. In between encoding trials, each face was presented a second time, without a name but with the caption “Judgment?” (JOL trials). For JOL trials, participants were asked to provide judgments about their confidence in being able to recall the name of the face several minutes later on a scale from 1 (i.e., *I am absolutely sure that I will not retrieve this name at a later memory test*) to 4 (i.e., *I am absolutely sure that I will retrieve this name at a later memory test*). During retrieval, each face was presented another time with the caption “Name?” (recall trials). For recall trials, the previously studied target name had to be retrieved. An illustration of the task procedure is depicted in Fig. 1. In order to match the response mode during JOL and cued recall, all responses were provided verbally and recorded using a MR-compatible headset and Adobe Audition 1.5.

### Imaging procedure

Using an event-related design, participants were presented with a series of pseudorandomly intermixed encoding, JOL, and cued recall trials. Stimuli were delivered using the Presentation software package (Version 10.1; Neurobehavioral Software, http://www.neurobs.com), as well as an MRI compatible audio-visual system consisting of a headset with included microphone and video goggles (VisuaStim XGA, Resonance Technology, Inc., http://www.mrivideo.com/). The entire experiment was conducted within the MRI scanner. Encoding and JOL trials were presented for 3000 ms, while recall trials lasted for 4000 ms. The time interval from an encoding trial to the corresponding JOL trial varied from 2 to 30 seconds (see also Supplementary File S1). The experiment was subdivided into seven blocks (see Fig. 1). Each of the 60 stimuli was assigned to one of the first six blocks. Each of these first six blocks contained ten face-name-pairs which had to be studied and provided with JOLs. Starting from the second block, and lasting until the seventh block, recall trials corresponding to encoding trials of the previous block were presented in the second part of each block. In order to arrange a balanced experimental design, filler items consisting of unfamiliar face-name pairs which were presented as study and JOL trials but not as recall trials were added, comprising five fillers in the second part of the first block and ten fillers in the first part of the seventh block.

All trials were presented at pseudorandomized, variable interstimulus intervals (ISI) between 2400–4200 ms. Null events consisting of a black cross in the center of a white screen, were presented for randomized, variable intervals between 2400–4200 ms within each item block. For the participants, null events were indistinguishable from baseline (see Fig. 1). All stimuli were presented again in a second functional run in order to additionally test for repetition effects, for instance, repetition suppression effects [19], or repetition priming effects on memory [20].

As the present report is focused on identifying the neural correlates underlying metamemory, the analyses were restricted to data from the first run, when critical encoding as well as monitoring occurred.

### MRI acquisition

All scanning was performed on a 1.5T Philips MRI scanner (Gyroscan Intera, Philips Medical Systems, Best, The Netherlands) using standard gradients and a circular polarized phase array head coil. For each of the two experimental sequences, a series of 538 T2*-weighted axial EPI volumes were acquired (repetition time (TR): 2800 ms, echo time (TE): 50 ms, number of slices (NS): 31 slices, slice thickness (ST): 3.5 mm, interslice gap (IG): 0.35 mm, matrix size: 64×64, field of view (FOV): 240×240 mm, voxel size: 3.75×3.75×3.8 mm, flip angle (FA): 90°). In between the two functional runs, an anatomical scan was acquired for anatomical localization using a high-resolution T1-weighted 3D-sequence consisting of 160 transversal slices (TR = 8210 ms, TE = 3800 ms, FoV = 256×256 mm, ST = 1.0 mm, interslice gap = 0.1 mm, FA = 8°).

### Data Analysis

FMRI data preprocessing was carried out using FEAT (FMRI Expert Analysis Tool) Version 5.98, part of FSL (FMRIB's Software Library, www.fmrib.ox.ac.uk/fsl) [21], [22]. The following preprocessing steps were applied: motion correction using MCFLIRT [23]; the mean absolute subjectś motion was 0.72 (s.d. = 0.4; range: 0.2–1.9), the mean relative value was 0.09. Slice-timing correction using Fourier-space time-series phase-shifting; non-brain removal using BET [24]; spatial smoothing using a Gaussian kernel of FWHM 8 mm; grand-mean intensity normalisation of the entire 4D dataset by a single multiplicative factor; highpass temporal filtering (Gaussian-weighted least-squares straight line fitting, with sigma = 50.0 s).

Afterwards, registration to MNI standard space images was carried out using FLIRT [23]. The first-level analyses for individual participants and second-level group analysis were performed using SPM5 (Wellcome Department of Cognitive Neurology, London, UK; www.fil.ion.ucl.ac.uk) implemented in MATLAB 7.0.4 (The MathWorks Inc., Sherborn, Massachusetts).

For each participant, the responses were sorted according to memory prediction (R = will remember; F = will forget) and actual memory outcome (H = hit; M = miss). Hits referred to the retrieval of the correct name, while misses were pooled over all trials for which either the wrong or no name was given. Thus, RH denotes an item which was successfully recalled after a “will remember” prediction, RM refers to an item which failed to be recalled after a “will remember” prediction, FH denotes an item which was successfully recalled after a “will forget” prediction and FM denotes an item which failed to be recalled after a “will forget” prediction.

Considering the different trial types employed in our design (ENC = encoding; JOL = judgment of learning; REC = recall), this sorting resulted in the following regressors:

- 3 regressors for trials provided with JOLs of 3 or 4, which were later successfully recalled (study RH, JOL RH, recall RH),
- 3 regressors for trials with JOLs of 1 or 2, which were later remembered (study FH, JOL FH, recall FH),
- 3 regressors for trials with JOLs of 3 or 4, which were forgotten (study RM, JOL RM, recall RM),
- 3 regressors for trials with JOLs of 1 or 2, which were forgotten later (study FM, JOL FM, recall FM).

The hemodynamic response for each of the 12 regressors was modeled by a canonical hemodynamic response function (HRF) and its temporal derivative. The temporal derivative was included in the model to account for the residual variance resulting from small temporal differences in the onset of the hemodynamic response, which is not explained by the canonical HRF alone. The functions were convolved with the event-train of stimulus onsets to create covariates in a general linear model. Parameter estimates for the HRF regressor were calculated from the least mean squares fit of the model to the time series. Parameter estimates for the temporal derivative were not further considered in any contrast. An SPM5 random-effects group analysis was performed by entering parameter estimates for all conditions into a within-subject one-way ANOVA, in which subjects are treated as random variables. If not noted otherwise, we used a threshold of p<0.001 uncorrected and an extent of at least 5 contiguous voxels for all contrasts. In order to perform a small volume correction (SVC) analysis [25], we used the peak coordinates of the contrasts of actual encoding success (corresponding to the “successful memory formation” contrast in our study) and predicted memory success (corresponding to our “encoding preceding predicted memory” contrast) from the study of Kao and colleagues [11], to define a priori regions of interests (ROI). These ROIs were employed for the SVC analysis in SPM5 at a p-value of 0.05 uncorrected and were considered as being significant if the corresponding voxelwise p value was less than 0.05 corrected for multiple comparisons across the ROI.

Signal change was analyzed by averaging activity within a sphere with a radius of 3 mm around the peak coordinate of interest (http://marsbar.sourcefourge.net/) [26]. The mean percent signal change over a time interval lasting from 0–11.2 s after stimulus onset was computed separately for each participant, brain region of interest and condition.

## Results

### Behavioral Data

The mean number of correctly recalled trials was 12.9 (s.d. = 3.8) and the mean number of misses was 47.1 (s.d. = 3.8). The mean number of “will remember” judgments was 17.3 (s.d. = 6.4), whereas “will forget” judgments were provided on average, for 42.7 trials (s.d. = 6.4). A 2x2 repeated measures analysis of variance (ANOVA) of performance with factors confidence (will remember versus will forget trials) and memory performance (hit versus miss trials) revealed a significant main effect of confidence (F_1,16_ = 165.8, *P*<0.001), indicating that participants gave more F-predictions than R-predictions. Furthermore, the interaction between performance and confidence (F_1,16_ = 17.15, *P*<0.001) was significant indicating that the JOLs predict memory performance (especially forgetting) to a certain degree. Post-hoc t-tests showed a highly significant difference between FM (mean number of trials = 37.12; s.d. = 7.3) and FH (mean number of trials = 5.5; s.d. = 2.37) (t_16_ = 14.94, *P*<10^−10^). As compared to this, RM (mean number of trials = 9.88; s.d. = 4.5) differed from RH (mean number of trials = 7.4; s.d. = 2.94), however at a lower level of significance (t_16_ = 2.54, *P*<0.05).

### Imaging Data

#### Successful memory formation (ENC_SM)

The comparison of activation during encoding of later hits to encoding activation for later misses, regardless of JOL prediction [(ENC RH + ENC FH)>(ENC RM + ENC FM)] revealed significant brain activation increases located in the right inferior frontal gyrus (RIFG; [40], [24], [11]), and in the left ACC [−8, 11, 34]. The analysis of the reverse contrast yielded no significant increases of blood-oxygen-level dependent (BOLD) response.

#### Encoding preceding predicted memory (ENC_PM)

For the comparison between all encoding trials which were subsequently assigned with a “will remember”-judgment versus those with a “will forget”-judgment, regardless of actual memory outcome [(ENC RH + ENC RM)>(ENC FH + ENC FM)], we found increased brain activation located exclusively in the left superior frontal gyrus (SFG; [−11, 63, 10]), LIFG [−45, 26, 19], and the middle frontal gyrus [−38, 38, −4]. The analysis of the reverse contrast showed no significant increases of BOLD response.

#### JOLs following successful memory formation (JOL_SM)

**Figure 2:**
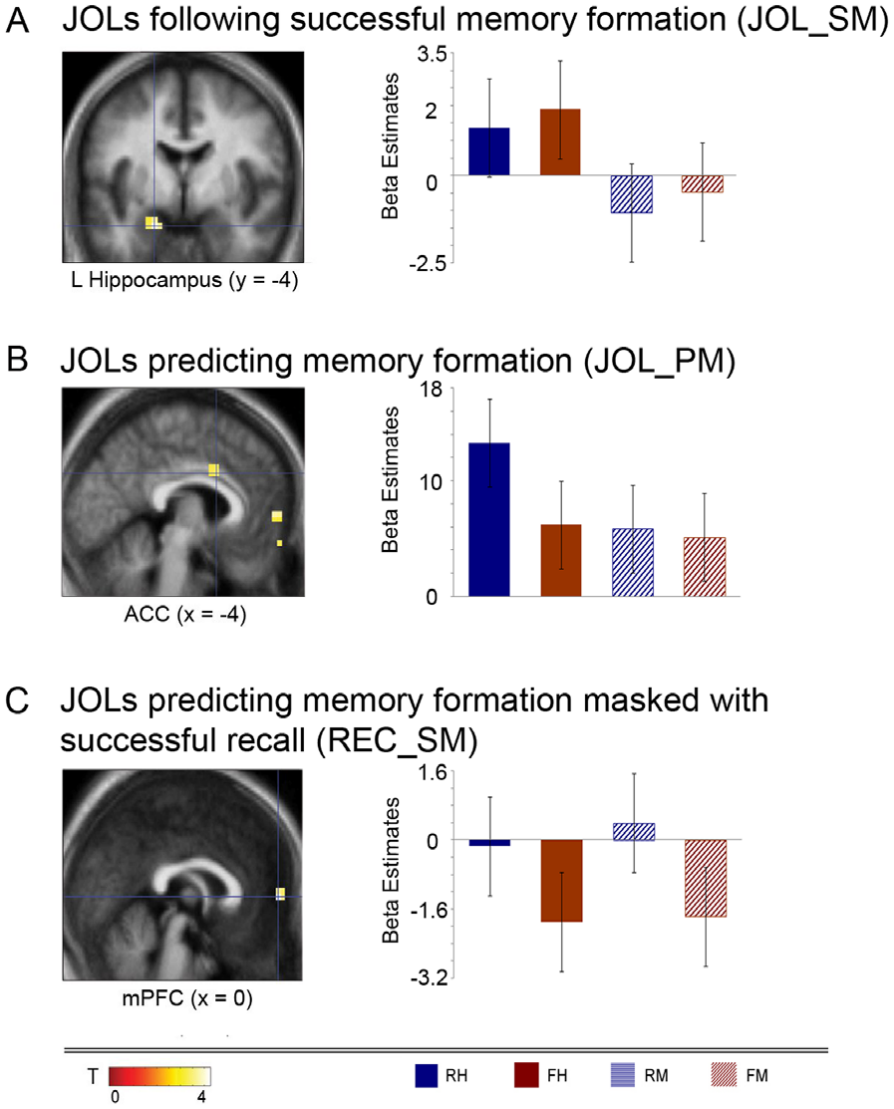
Statistical activation maps, and bar graphs depicting the parameter estimates per condition. Activation maps are overlaid onto the mean anatomical image across participants. Regions of interest (ROIs) defined from (a) JOLs following successful memory formation (JOL_SM) were located in the left MTL; (b) JOLs predicting memory formation were located in the ACC; (c) JOLs predicting memory formation (JOL_PM) masked with successful recall (REC_SM) was located in the mPFC. Coordinates are presented in Table 1.

We compared brain activation during metamemory judgments for later hits to metamemory judgments for later misses [(JOL RH + JOL FH)>(JOL RM + JOL FM)]. This contrast revealed activations of the right and left hippocampi (Fig. 2A) (MNI coordinates: right hippocampus, [19, −8, −26]; left hippocampus, [−19, −4, −24]; for an overview of all significantly activated regions in all contrasts, see Table 1). The reverse contrast (JOLs for later misses versus JOLs for later hits) did not reveal any significant activation increases.

**Table 1:** Brain areas associated with (a) successful memory formation and (b) encoding preceding predicted memory, (c) JOLs following successful memory formation, (d) JOLs predicting memory formation, (e) JOLs predicting memory formation masked with successful recall, and (f) JOLs predicing memory formation masked with successful recall corrected for cue recognition.

				MNI Coordinates				
Effect	Anatomical Region		BA	x	y	z	T-value	Cluster size
(a) **Successful memory formation (ENC_SM)**								
	Inferior Frontal	R	13	40	24	11	3.53	22
	Anterior Cingulate	L	32	−8	11	34	3.73	7
(b) **Encoding preceding predicted memory (ENC_PM)**								
	Inferior Frontal	L	46	−45	26	19	4.14	13
	Superior Frontal	L	10	−11	63	10	4.16	48
	Middle Frontal	L	47	−38	38	−4	4.39	11
(c) **JOLs following successful memory formation (JOL_SM)**								
	Hippocampus	L		−19	−4	−24	4.15	12
	Hippocampus	R		19	−8	−26	4.01	5
(d) **JOLs predicting memory formation (JOL_PM)**								
	Medial Frontal		11	0	56	4	4.25	19
	Orbital Frontal			0	56	−15	3.73	9
	Anterior Cingulate			−4	8	30	3.36	5
(e) **JOLs predicting memory formation (JOL_PM) masked with successful recall (REC_SM)**								
	Medial Frontal	L	10	−4	56	−7	4.25	11
	Superior Temporal	L		−53	−34	8	3.24	5
(f) **JOLs predicting memory formation (JOL_PM) masked with successful recall corrected for cue recognition**								
	Medial Frontal	L	10	−4	60	1	4.65	24
	Superior Temporal	L		−47	−38	12	3.29	5
	Anterior Cingulate	L	32	−3	38	21	3.27	5

Only cluster of five or more voxels and a significance of *P*<0.001 uncorrected are reported. BA, Brodmańs area; L, left; R, right.

#### JOLs predicting memory formation (JOL_PM)

As a next step, we compared activations during JOL predicting remembering to those predicting forgetting, irrespective of actual memory outcome [(JOL RH + JOL RM)>(JOL FH + JOL FM)]. We found increased activation located in medial prefrontal cortex (mPFC; [0, 56, 4]), orbital frontal cortex (OFC; [0, 56, −15]), and in the anterior cingulate cortex (ACC; [−4, 8, 30]) (Fig. 2B). The analysis of the reverse contrast comparing all JOLs predicting memory failure to all JOLs predicting memory success yielded no significantly increased activation.

#### Common neural correlates of predicting memory formation (JOL_PM) and successful recall (REC_SM)

To test for the hypothesis that retrieval operations might be involved in the process of forming JOLs, we aimed to delineate common neural correlates of JOL_PM and successful memory recall (REC_SM: increased brain activation during recall for hits versus misses). To this end, we masked activations during prediction of successful memory formation [(JOL RH + JOL RM)>(JOL FH + JOL FM)] inclusively by activations of successful memory recall (see Fig. S1), regardless of JOL prediction. The mask used was derived from the contrast [(REC RH + REC FH)>(REC RM + REC FM)] at an uncorrected p-value of 0.05. This analysis revealed that the left mPFC [−4, 56, −7] and the left superior temporal gyrus (STG; [−53, −34, 8]) are critical both for predicting successful memory performance and successful memory recall (Fig. 2C). We also inclusively masked prediction of successful memory [(JOL RH + JOL RM)>(JOL FH + JOL FM)] by activations during unsuccessful recall [(REC RM + REC FM)>(REC RH + REC FH)] (uncorrected mask p-value = 0.05). This analysis yielded no significant increase in BOLD-response. Since brain activation related to cue recognition might contribute to the shared pattern of activation found in the contrast of JOLs predicting memory formation and successful retrieval, we performed an additional analysis aiming to dissociate between activation associated with cue recognition of the face and cued name recall. This analysis applied the following logic: when subjects stated a name during retrieval after onset of the face cue, it can be assumed that they identified the face cue as familiar, i.e., as previously seen, regardless of whether they responded with the correct or incorrect name. In order to dissociate cued name recall from cue recognition, we thus sorted the responses during the recall phase into the following conditions: a) no name was recalled [r1], b) the incorrect name was recalled [r2], and c) the correct name was stated [r3].

We then build the following new mask for successful recall corrected for cue recognition: correct name recall versus incorrect name recall [REC Rr3>REC Rr2]. This contrast should delineate the neural network involved with successful recall of the name while minimizing the contribution of face recognition, because both conditions should involve this process.

Inclusive masking (at an uncorrected p-value of 0.05) of JOLs predicting memory formation by the new mask revealed a shared pattern of activation in mPFC [−7, 60, 0] which, by use of a small volume correction analysis (at a p-value of 0.05), could be shown to overlap with the mPFC activation cluster observed for masking JOLs predicting memory formation with the original mask (see above) within a sphere of 10 mm (p>0.05, FWE corrected). Increased brain activations of the new mask itself, were located in the left MFG [−8, 60, −12], the right SFG [15, 60, −8], the bilateral parahippocampal gyri (PHG, right [23, −15, −15], left [−15, −19, −19]), right BA 11 [30, 49, −11] and left BA 11 [−38, 56, −11], midbrain [4, −30, −11], and LIFG [−38, 30, 4]. The opposite contrast did not reveal any significant activation increase.

In order to identify differences between the neural correlates of JOLs and memory encoding, we exclusively masked JOL-related contrasts by encoding-based contrasts and vice versa, at a mask threshold of p<0.05 uncorrected (minimum-cluster-size of 5 voxels). The choice of a very liberal threshold for the exclusive mask results in a more conservative masking procedure.

#### ENC_SM and JOL_SM

Exclusively masking brain activation during JOLs following successful memory formation (JOL_SM) by activation related to successful memory formation during encoding (ENC_SM) revealed significant activations in bilateral hippocampi (right hemisphere: [19, −8, −23]; left hemisphere: [−19, −4, −27]). On the other hand, exclusively masking brain activation related to successful memory formation by activation during JOLs predicting memory formation showed increased BOLD response in the RIFG [40], [23], [13] and the left ACC [−9, 11, 28].

#### ENC_PM and JOL_PM

Exclusive masking of JOLs predicting memory formation (JOL_PM) by activations for encoding trials preceding predicted memory (ENC_PM) showed significant activation increases of the MFG [4, 64, 8] and the OFG [−4, 56, −7], while exclusive masking encoding preceding predicted memory by activation for JOLs predicting memory formation. The reversed analysis revealed increased BOLD responses of the left SFG [−19, 49, 23], the LIFG [−49, 23, 0], and the left middle frontal gyrus [−49, 24, −19].

## Discussion

The present study was conducted to reveal the neural correlates of JOLs, aiming to disentangle brain networks involved with memory encoding, metamemory judgments, and memory recall. Specifically, we were interested in investigating whether retrieval processes are critical for JOLs, as has been suggested before [8], [14], [15]. For instance, the monitoring-retrieval view hypothesizes that metacognitive assessments of onés own memory performance might rely on the monitoring of information about the critical item, which is retrieved from memory [14]. Similarly, the monitoring-dual-memories explanation proposes retrieval processes as a pre-condition for JOLs [27]. Thus, we examined common neural networks involved with the formation of JOLs and successful memory retrieval to support these propositions by functional imaging data. In previous imaging studies, JOLs were acquired at the time of memory encoding [11]. Therefore the neural correlates of both processes could not be separated from each other. In contrast, in our study, JOLs were temporally separated from encoding to allow for a genuine analysis of predictions from the monitoring-retrieval theory, as a recent electroencephalography study showed that JOLs do not only represent encoding operations [28]. In order to identify regions in which activations were not shared between encoding and JOL, we exclusively masked brain activation during memory encoding with activation during JOLs and vice versa. We found brain regions for JOLs following successful memory (JOL_SM) which dissociated from those for successful memory formation (ENC_SM). Also, brain regions for JOLs predicting memory formation (JOL_PM) dissociated from those found for encoding preceding predicted memory (ENC_PM).

The current study represents further evidence in the fMRI investigation of JOLs, since its experimental design provides a means to test metamnemonic operations beyond the encoding phase [11]. The finding of a common neural network which is recruited both during JOLs and successful retrieval in the present study might be specific to spaced JOL. The behavioral literature suggested that cognitive processes underlying memory predictions provided simultaneously with learning differ from those underlying predictions given after a delay (for an overview of the effects of delayed memory predictions, see [9]): While immediate JOLs mainly rely on information from short-term memory including biasing interferences, spaced JOLs were found to rely on information from long-term memory [27]. The information recruited in forming delayed JOLs can be regarded as a kind of retrieval practice which is similar to retrieval during a subsequent memory test [14]. Since retrieval practice might involve rehearsal of the target name [62], or refreshing of visual information of the face cue [63], it might lead to more vivid memory traces and therefore account for increased monitoring accuracy of delayed as compared to immediate JOLs. Our findings indicate that differential operations are executed during encoding and metamnemonic JOLs and therefore justify a separation of JOL phase from encoding phase.

### Successful memory formation

In accordance with findings of Kao and colleagues (see their contrast named “actual memory success”, [11]: Table 1, p. 1778) our analyses revealed the right lateral PFC to be related to successful memory formation (ENC_SM). Studies of declarative memory have demonstrated frontal lobe activity to be critically involved in memory formation and retrieval [29], [30], [16] and to be specifically related to the selection and organization of incoming information [31]. The RIFG has been reported to be crucial for novelty detection of task-relevant features [32], but also for updating of corresponding action intentions [33]. Thus, the contribution of the RIFG might be discussed as follows: the image of a single face (i.e., the stimulus cue per se) might lead to an unreliable judgment. Therefore, more semantically related information about the cue is searched for [34] and this additional information will improve the reliability of the JOL.

Furthermore, our data revealed an involvement of the ACC in successful memory formation. The ACC is known for performance monitoring [35], and integration of detected conflicts and attentional control mechanisms [36]. Thus, this activation most probably is related to attentional processes during memory encoding in our quite demanding memory task.

### Encoding preceding predicted memory success

With regard to encoding preceding predicted memory success (ENC_PM), our data revealed increased activation of the left PFC. This finding corresponds with findings from Kao and collaborators (see the contrast named “predicted memory success”, [11]: Table 1, p. 1778). For the face and name stimuli employed in the present study, semantic processing [37] might be especially important to integrate the facial stimuli into a coherent semantic memory representation [13], for instance, “the nose of this girl Susan reminds me of the nose of Barbara Streisand” could result in a memory representation such as “Barbara Streisand – nose – Susan”. Subsequently, the memory representation with the strongest predictive index for recallability will be selected as basis for JOLs [31]. Furthermore, increased activation of regions of the left SFG has been shown to refer to higher cognitive functioning [38], to monitoring and manipulation of information [39], and especially to self-awareness processes [40]. In a number of studies, areas located in the SFG and in the middle frontal gyrus have been identified as being critical components of WM processing [41]–[44]. In order to establish a new memory representation, each single feature needs to be maintained for the integration into a representational network. Thus, encoding processes in left lateral PFC regions should be important preparatory operations for later JOLs.

### JOLs following successful memory formation

In the present study, we separated JOL from encoding trials in order to disentangle the neural and cognitive processes during JOLs from those during encoding. For JOLs following successful memory formation (JOL_SM), we found increased brain activation of the bilateral hippocampi. Previous studies have shown hippocampal activity to be critical for associative memory recall [45] and cued recall of paired associates [46], especially for the retrieval of face-name pairs [47]. Thus, our finding of hippocampal activation during JOLs might imply the execution of recollection operations during metacognitive judgments. Alternatively, the hippocampal activation might be explained by an additional re-learning, or establishing of recruited information into a memory representation, during JOLs. The latter assumptions could not be sufficiently operationalized by our paradigm, but should be an interesting topic for a further experiment.

### JOLs predicting memory formation

We found that JOLs predicting memory formation (JOL_PM) are associated with increased activation of medial and orbital frontal regions as well as the ACC. Increased activation of the mPFC has been associated with self-referential processes and self-knowledge [48], but also with memory predictions and self-relevant judgments [49]. Since metamnemonic judgments refer to knowing about own knowledge, we interpret mPFC activation increases in terms of reflecting introspective operations [11].

Furthermore, our data revealed increased brain activation of the OFC, which has been shown to be involved in integrative processing of sensory information [50], in decision-making [51], and in executive functions such as the regulation of goal-directed behavior [52]. With respect to JOLs predicting memory formation, the contribution of the OFC might represent effort to integrate visuosensory information of the presented face stimuli into the procedure of making a decision about whether or not the correct name will be remembered subsequently. An increased activation of the ACC in the context of predicting memory performance might reflect its engagement in general performance monitoring [53], [54], or in managing the attentional focus on input essential for JOLs [36].

### Recollection during formation of JOLs

As it has been suggested that JOLs are based on retrieval processes, we masked activation for JOLs predicting successful memory with actual retrieval success to identify common neural networks involved with the formation of JOLs and with memory recall. This analysis showed that the mPFC is involved with both successful recall and prediction of memory performance. Specifically, the mPFC might be engaged in adjustments of subsequent behavior [55], as well as in monitoring of retrieval outcome but it might also imply that metamnemonic processes are involved during retrieval [56]. Common neural correlates of JOLs and memory recall were also found in the left superior temporal gyrus, a region known for phonological processing of speech dependent contents [57], [58]. Since we suggest that monitoring is associated with retrieval processes, this might reflect introspective speech to self-evaluate the output of the retrieval attempt which again supports the process of forming a JOL. Since memory recall can be described as a core retrieval process with accompanying pre-recall and post-recall monitoring and control processes, associative memory recall might involve a preprocess related to the identification of a face cue which again would be a precondition for initiating retrieval [59]. Having recognized the face as familiar would also effect the level of JOLs [60], [61]. In order to address the question whether brain activation related to the successful face cue recognition may contribute to the common pattern of activation shared by JOLs predicting memory formation and successful recall, we performed an additional analysis aiming to dissociate the activation associated with cue recognition or familiarity of the face from activation related to cued name recall. When inclusively masking JOLs predicting memory formation using the mask consisting of the contrast of correct name recall versus incorrect name, we again observed a shared activation located within mPFC in close vicinity to the activation reported for the original mask. As the mask used in this analysis should minimize the influence of cue recognition, we suggest that the activation in mPFC is related to name recall itself instead of pre-recall monitoring processes.

### Conclusions

The present study investigated the neural correlates of metamnemonic monitoring and associated cognitive processes. First, by temporally separating memory encoding and JOLs, we were able to show that distinct activations are involved with both processes. Furthermore, our analyses of successful memory formation (ENC_SM) and encoding preceding predicted memory success (ENC_PM) revealed increased brain activation of regions which correspond to those in the study by Kao and colleagues [11], thus replicating their findings for a paired associates design.

Concerning brain activation during JOLs, our data revealed three core results: 1) JOLs following successful memory formation (JOL_SM) were associated with increased activation of the bilateral hippocampi, indicating that these JOLs might be based on successful recall of associative memory representations 2) JOL predicting memory formation (JOL_PM) was associated with activation in medial and orbital frontal cortices, and in the ACC, presumably reflecting introspective operations during metacognitive judgments. 3) The medial prefrontal cortex was activated both during successful memory recall and during JOLs predicting memory success, supporting the hypothesis that retrieval attempts provide a basis for JOLs. We interpret this finding as indicating that JOLs actually incorporate retrieval operations, for instance, covert rehearsal.

## Supporting Information

Figure S1 **Overview of all additional regions showing increased brain activation during the retrieval phase for hits as opposed to misses (Fig. S1A–D).**
(TIF)

File S1 **Methods: The issue of whether a delay increases the accuracy of JOLs.**
(DOC)
