# Supplementary material for: Monitoring the Mind: The Neurocognitive Correlates of Metamemory
Source: PLoS One. 2012 Jan 5;7(1):e30009. doi: 10.1371/journal.pone.0030009 (PMC3252366; doi:10.1371/journal.pone.0030009)
Supplement: File S1 — Methods: The issue of whether a delay increases the accuracy of JOLs. (DOC) [file pone.0030009.s002.doc]

**Supplementary File S1**

***Methods:* The issue of whether a delay increases the accuracy of JOLs**

A further interesting issue in the behavioral research on metamemory refers to the influence of a time lag between study and JOL. Some studies found memory judgments which were provided after a delay to be significantly more accurate than judgments given immediately [1]. Monitoring of long-term memory without noise as opposed to monitoring of short-term memory at the time of delayed JOLs was discussed to account for this increase in metamemory accuracy [2]. Other researcher suggested that the so-called “delayed JOL effect” is based only on retrieval practice [3]. Altogether, sufficient evidence for the existence of systematic effects due to longer intervals between learning and JOL is lacking in the literature. Since no significant differences between accuracy of immediate versus delayed judgments were observed in our behavioral data (t_16_= 1.71, *P* > 0.1), we collapsed both types of JOLs to yield adequate numbers of trials per condition.

***REFERENCES:***

1. Nelson TO, Dunlosky J (1991) The delayed-JOL effect: When delaying your judgements of learning can improve the accuracy of your metacognitive monitoring. Psychol Sci 2: 267-270.

2. Dunlosky J, Nelson TO (1992) Importance of the kind of cue for judgments of learning (JOL) and the delayed-JOL effect. Mem Cognition 20: 374-380.

3. Spellman BA, Bjork RA (1992) When predictions create reality:  Judgments of learning may alter what they are intended to assess.  Psychol Sci 3: 315-316.
